# ADRB2 Regulates the Proliferation and Metastasis of Gastrointestinal Stromal Tumor Cells by Enhancing the ETV1-c-KIT Signaling

**DOI:** 10.1155/2023/6413796

**Published:** 2023-02-03

**Authors:** Sijun Chen, Feijing Wu, Jiaxuan Zhang, Jianwei Zhu, Xiaorong Zhou, Xiaofei Zhi

**Affiliations:** ^1^Department of General Surgery, Affiliated Hospital of Nantong University, Nantong 226001, Jiangsu, China; ^2^Department of General Surgery, The Second Affiliated Hospital of Jiaxing University, Jiaxing 314000, Zhejiang, China; ^3^Department of General Surgery, The Second Affiliated Hospital of Fujian Medical University, Quanzhou 362000, Fujian, China; ^4^Department of Trauma Center, Affiliated Hospital of Nantong University, Nantong 226001, Jiangsu, China; ^5^Department of Immunology, Nantong University, Nantong 226001, Jiangsu, China

## Abstract

**Background:**

Gastrointestinal stromal tumor (GIST) originates from a pacemaker cell, the Cajal cell. However, little is known about the cancer neuroscience in GIST. In this study, we aimed to elucidate the clinical and biological roles of adrenoceptor beta 2 (ADRB2) in GIST.

**Methods:**

Immunohistochemistry was used to evaluate the expression of ADRB2 in GIST tissues. The biological effects of ADRB2 on GIST cell proliferation, migration, invasion, and apoptosis were explored using Cell Counting Kit −8, plate colony formation assay, transwell invasion assay, and flow cytometry. We also explored the growth and metastasis of xenograft tumors in nude mice. Western blotting was used to quantify protein expression and phosphorylation.

**Results:**

ADRB2 is generally highly expressed in GIST. High ADRB2 expression was significantly associated with risk level, tumor size, mitotic count, and metastasis. Overexpression of ADRB2 promoted GIST cell proliferation, migration, invasion, and apoptosis, while silencing ADRB2 expression showed the opposite effects. Furthermore, we found that silencing endogenous ADRB2 inhibited GIST progression and metastasis in nude mice. ADRB2-induced ETV1 upregulation enhanced the activation of c-KIT.

**Conclusion:**

ADRB2 plays an important role in the proliferation and metastasis of GIST and is expected to be a potential target for the treatment of GIST.

## 1. Introduction

Gastrointestinal stromal tumor (GIST), which is the most common mesenchymal tumor of the gastrointestinal tract, originates from the interstitial Cajal cells or their precursors [1, 2]. GIST usually occurs in the stomach (60%–70%) and small intestine (20%–25%) [3]. People between the ages of 50 and 70 are high-risk individuals [4]. At present, the main treatment methods for GIST are surgical resection and biological therapies, such as imatinib. However, GIST patients with recurrence and metastasis still lack effective treatments [5, 6]. Therefore, it is imperative to explore the biological mechanism, find new biomarkers, and provide new strategies for the diagnosis and treatment of GIST.

Multiple studies have shown that tumor progression is associated with chronic stress [7–10]. The sympathetic nervous system plays an important role in chronic stress [11]. Its nerve fibers act on adrenergic receptors by releasing catechol neurotransmitters to regulate cellular function [12, 13]. The gastrointestinal tract is often injured under stress in the human body [14], such as by stress ulcers. Adrenoceptor beta 2 (ADRB2) is an important member of seven transmembrane G protein-coupled receptors that can be activated by *β*-agonists such as epinephrine, norepinephrine, and isoproterenol [15]. Recent studies have found that ADRB2 signaling can regulate a variety of cells in the tumor microenvironment and activate cancer-related signaling pathways [16]. However, the role of ADRB2 signaling in gastrointestinal stromal tumors remains unclear.

In this study, we found that patients with high ADRB2 expression had a worse prognosis. We also showed that ADRB2 could promote GIST cell growth and apoptosis both in vitro and in vivo. In addition, ADRB2 enhances the ETV1-c-KIT signaling [17] by inducing ETV1.

## 2. Materials and Methods

### 2.1. Cell Lines and Cell Culture

The GIST-882 human GIST cell line was obtained from Lonza, and the GIST-T1 human GIST cell line was purchased from Cosmo Bio. The cells were incubated in RPMI 1640 (Gibco, NY, USA), supplemented with 10% FBS (Invitrogen, Life Technology, CA, USA) and 1% penicillin-streptomycin (Sigma, St. Louis, USA). All cell lines were cultured at 37°C with 5% CO_2_ in an incubator.

### 2.2. Patients and Tissue Microarray (TMA)

The GIST tissue samples used to construct the tissue microarray were collected from 122 GIST patients who underwent radical resection at the Affiliated Hospital of Nantong University (Nantong, China) from 2010 to 2018. Besides, we also collected 22 liver metastatic samples from advanced GIST patients who underwent palliative surgery. All tissue samples were identified by HE staining and immunohistochemical staining (CD117, CD34, SMA, and Desmin4). Immunohistochemical analysis and scoring were performed as previously described [18]. All participants obtained informed consent, and this study was approved by the Ethical Committee of the Affiliated Hospital of Nantong University. All experiments strictly followed the principles of the Declaration of Helsinki.

### 2.3. qRT-PCR

Total cellular RNA from human tissues was extracted using TRIzol (Takara, Shiga, Japan), and cDNAs were generated using PrimeScript RT reagent (Takara, Dalian, China). Subsequently, qRT-PCR was performed using the Power SYBR Green PCR master mix (Applied Biosystems, Foster City, USA) according to the manufacturer's instructions. The results were normalized to the levels of GAPDH. The primers used were as follows: ADRB2: forward 5′-AGAGCCTGCTGACCAAGAAT-3′ and reverse 5′-TAGCAGTTGATGGCTTCCTG-3′; *β*-actin: forward 5′-TCACCCACACTGTGCCCATCTACGA-3′ and reverse 5′-CAGCGGAACCGCTCATTGCCAATGG-3′.

### 2.4. Western Blotting

Total protein extraction from cells using lysis buffer (absin, Shanghai, China). Cellular proteins were separated by electrophoresis on a 10% SDS-polyacrylamide gel and then transferred to a polyvinylidene fluoride membrane by electroblotting. Following blocking with a 5% nonfat dry milk solution for 2 hours, the membrane was incubated overnight at 4°C with the appropriate primary antibody (1 : 1000 dilution). Finally, the membrane was incubated with the corresponding secondary antibody (1 : 5000 dilution) for 2 h at room temperature. The anti-c-KIT antibody was obtained from Invitrogen. Anti-p-ERK (Y204), anti-ERK, and anti-GAPDH were obtained from Cell Signaling Technology. Anti-ETV1 was obtained from Abcam. Secondary antibodies were purchased from Absin.

### 2.5. Cell Proliferation and Colony Formation Assay

Cells were seeded into 96-well plates at a density of 2000 cells/well and stained with Cell Counting Kit-8 (CCK-8, Dojindo, Kumamoto, Japan) according to the manufacturer's instructions. Cell viability was detected by measuring the absorbance at 450 nm. Cells (1,000 cells/well) were seeded into complete medium in 6-well plates and cultured in the incubator for 14–20 days. Then, the cells were fixed with methanol and stained with a crystal violet solution. After drying, the stained colonies in the 6-well plate were photographed and counted.

### 2.6. Wound Healing Assay

Cells were seeded in FBS-free medium in 6-well plates at a concentration of 5 × 10^5^ cells per well. After the cells have filled the entire area, a yellow pipette tip was used to make a horizontal wound. PBS was used to wash the cells to remove the floating cells. Following that, the wounds were photographed using an inverted microscope at 0 and 24 h of incubation.

### 2.7. Transwell Invasion Assays

Transwell assays were conducted to assess cell invasion and migration capacity. Matrigel matrix (Corning, MA, USA) was placed in the upper chamber. Then, the cells were seeded into FBS-free medium in the upper chambers. A complete medium with 20% FBS was added to the lower chamber as an inducer. After 24 hours, cells were fixed with methanol and stained with a crystal violet solution. Cells on the upper surface were erased, and cells on the lower surface were photographed and counted.

### 2.8. Cell Apoptosis Analysis

Annexin V-FITC apoptosis assay kit (Absin, Shanghai, China) and flow cytometry were used to analyze cell apoptosis. Cells were stained with Annexin V-FITC and propidium iodide following the manufacturer's instructions. Subsequently, the cells were examined on a flow cytometer (BD Biosciences).

### 2.9. Cell Transfection

Full-length Small hairpin RNAs (shRNA) for ADRB2 and their corresponding negative controls were obtained from Shanghai Genechem Co. Ltd (Shanghai, China). Sequence for shRNA1 is as follows: 5′ GGACCTGAGTCTGCTATATTT 3′; Sequence for shRNA2 is as follows: 5′ AGGTACTGTGCCTAGCGATAA 3′. Sequence for negative control is as follows: 5′ GTTCTCCGAACGTGTCACGT 3′. GIST cells were incubated with retroviral in six-well plates according to the manufacturer's instructions. Stable cell lines expressing ADRB2 or those with ADRB2 silenced were selected using puromycin.

### 2.10. Immunohistochemistry

Tissue sections were baked at 60°C for 1 h, followed by immersion into xylene, 100% ethanol, and then decreasing concentrations of ethanol. Antigen retrieval was done with a citric acid buffer. Sections were then blocked with 5% FBS and stained with first antibodies against ADRB2, c-KIT, and Ki67, followed by incubation with biotinylated secondary antibody and visualized by the standard avidin-biotinylated peroxidase complex method. Lastly, the tissue was counterstained with hematoxylin.

Immunoreactivity scores were assessed by the intensity of staining and percentage of positive area: − (negative staining or weak intensity under 10% aera), + (weak intensity between 10% and 100% or moderate intensity under 10%), ++ (moderate intensity over 10% or strong intensity under 90% aera), and +++ (strong intensity over 90% aera). Tissues with ++/+++ were considered as high expression, while tissues with −/+ were considered as low expression.

### 2.11. Animal Studies

BALB/c nude mice were provided by the Laboratory Animal Center of Nantong University. All animals were raised under pathogen-free conditions and had free access to water and food. GIST cells were injected subcutaneously into the axilla of nude mice (approximately 10^6^ cells in 100 *μ*l PBS per mouse). Tumor fragments were transplanted into the gastric wall of nude mice as previously described [18]. Tumor volume was calculated every 6 days using the following formula: Volume = (width^2^ × length)/2. The experimental protocols were approved by the Animal Care and Use Committee of the Laboratory Animal Center of Nantong University.

### 2.12. Statistical Analysis

All experiments were repeated at least three times. The data were expressed as the mean ± SD. Two-tailed Student's *t*-test was employed to calculate the difference between two groups. The *χ*^2^ test was performed to determine the association between patient clinical characteristics and ADRB2 expression. Spearman's correlation was used to analyze the expression of ADRB2 and c-KIT in tumor tissue samples. All statistical analyses were performed using GraphPad Prism 8.0 software and SPSS 25.0 statistical software. Two-tailed *P* < 0.05 was considered statistically significant.

## 3. Results

### 3.1. The Expression of ADRB2 Correlates with the Prognosis of Patients

**Figure 1:**
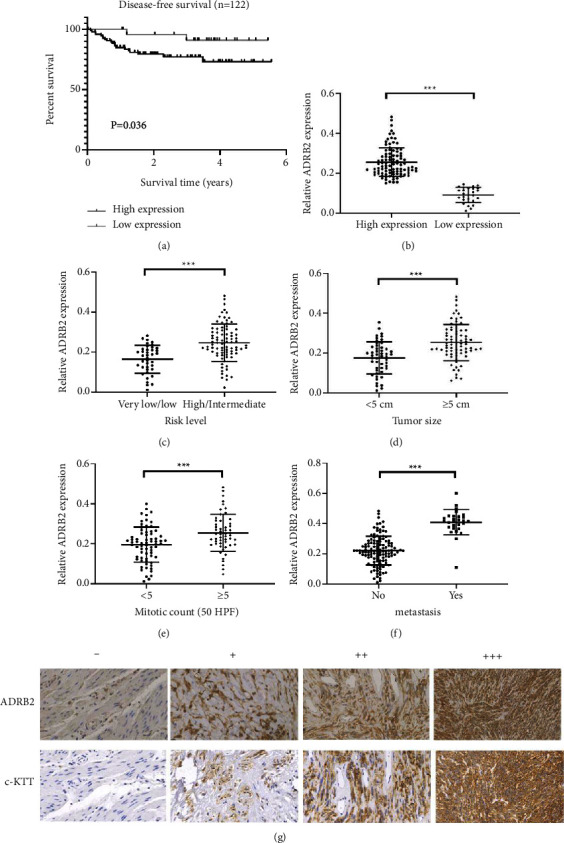
High ADRB2 expression is associated with prognosis in patients with gastrointestinal stromal tumors. (a) Kaplan–Meier disease-free survival curves for 122 patients with gastrointestinal stromal tumor stratified by high and low expression of ADRB2. (b) Real-time PCR was used to detect the mRNA levels of ADRB2 in high immunoreactivity-score group compared to low immunoreactivity-score group. (c–f) The mRNA levels of ADRB2 correlate with risk level, tumor size, mitotic count and liver metastasis. (g) Representative staining of ADRB2 and c-KIT in GIST tissues. Magnification: 400×; ^∗∗∗^*P* < 0.001.

To explore the expression and clinical significance of ADRB2 in GISTs, we constructed TMAs from 122 GIST patients and examined the expression of ADRB2 by immunohistochemical staining. Among 122 GIST patients, 96 (78.7%) patients had high (++/+++) ADRB2 expression in tumor tissue. Kaplan–Meier survival analysis showed that patients with high ADRB2 expression had a worse prognosis (*P* = 0.036; Figure 1(a)). In addition, the expression levels of ADRB2 were related to risk level, tumor size, and nuclear mitotic count (Table 1). We also analyzed the differences of ADRB2 expression between radical resection samples and palliative resection samples. As shown in Table 2, high expression level of ADRB2 was significantly correlated with liver metastasis. Subsequently, we detected mRNA levels of ADRB2 in GIST frozen samples. The high immunoreactivity-score group showed significantly higher levels of ADRB2 mRNA compared to the low immunoreactivity-score group (Figure 1(b)). The mRNA levels of ADRB2 were also correlated with risk level, tumor size, nuclear mitotic count, and liver metastasis (Figures 1(c)–1(f)). These data suggested that ADRB2 may be associated with the progression of GISTs. Interestingly, we also found that ADRB2 expression showed a significant correlation with c-KIT levels (Figure 1(g)). Spearman's correlation analysis showed that ADRB2 expression was positively correlated with c-KIT expression in GIST tumors (*R*^2^ = 0.581; *P* < 0.001; Table 3).

### 3.2. ADRB2 Modulates GIST Cell Proliferation

**Figure 2:**
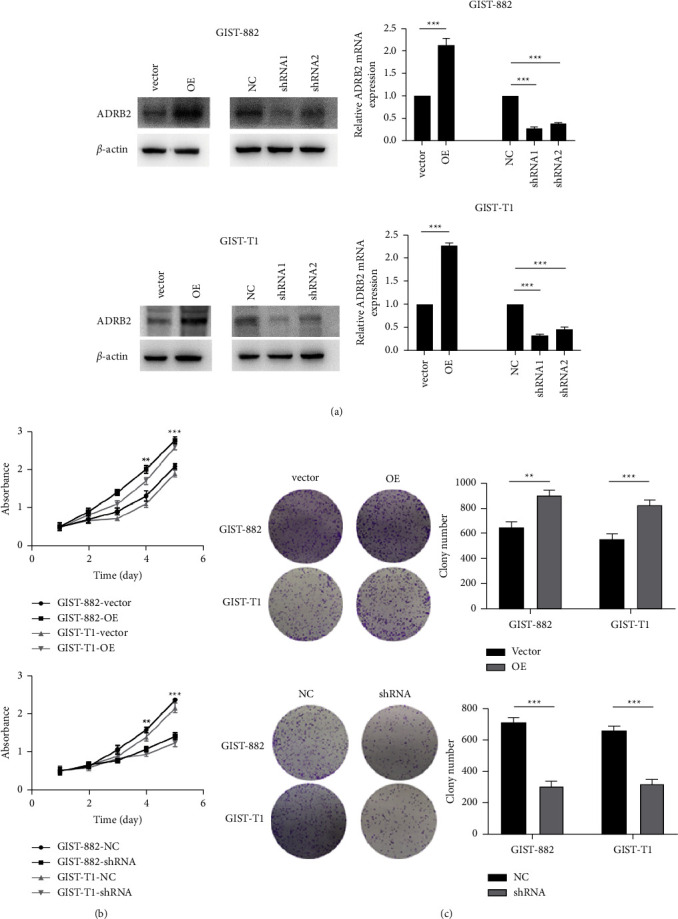
ADRB2 regulates tumor proliferation in gastrointestinal stromal tumors. (a) Western blot and real-time PCR confirmed overexpression and knockdown of ADRB2 in GIST-882 and GIST-T1 cells. (b, c) Cell proliferation abilities were detected by CCK-8 assay and colony formation assay. Data are presented as the mean ± SD. ^∗∗^*P* < 0.01; ^∗∗∗^*P* < 0.001.

To further explore the function of ADRB2 in GIST cells, we ectopically overexpressed ADRB2 in GIST-882 and GIST-T1 cells (Figure 2(a)). CCK-8 assays showed that overexpression of ADRB2 promoted the proliferation of GIST-882 and GIST-T1 compared with vector-transfected cells (Figure 2(b)). Colony formation assays also indicated the similar result to the CCK-8 assays (Figure 2(c)). Furthermore, GIST-882 and GIST-T1 cells were transfected with ADRB2-sh1, ADRB2-sh2, or negative control (shNC, Figure 2(a)). Because ADRB2-sh1 has a more pronounced effect, it was selected for the following experiments. Both CCK-8 and colony formation assays suggested that knockdown of ADRB2 significantly reduced the proliferation of GIST cells (Figures 2(b) and 2(c)). These results illustrate the critical role of ADRB2 in GIST cell proliferation.

### 3.3. ADRB2 Promotes GIST Cell Migration and Invasion

**Figure 3:**
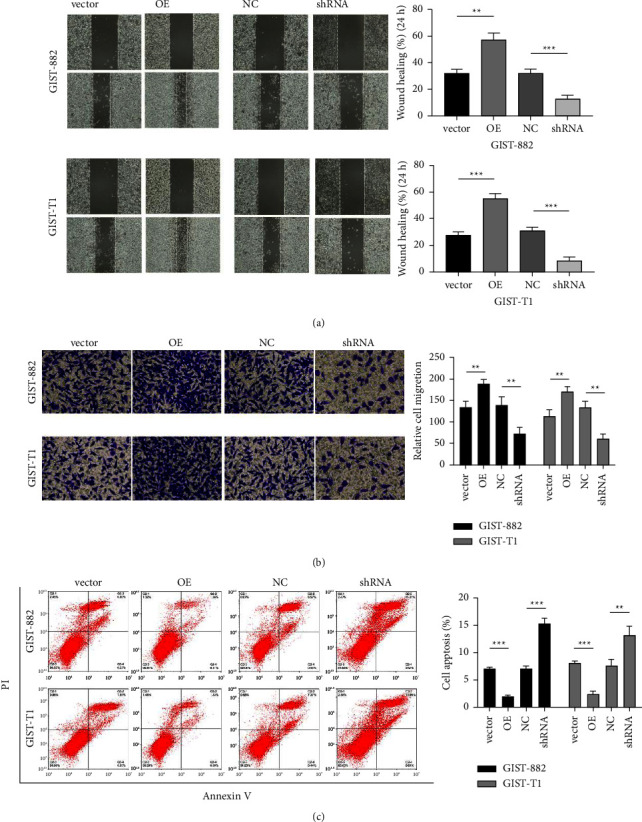
ADRB2 regulates GIST cell migration, invasion, and apoptosis. (a) Cell migration ability was determined by the wound healing assay; (b) transwell assay was used to determine cell invasion ability; (c) cell apoptosis was examined by staining with Annexin V/PI. Data are presented as the mean ± SD. ^∗∗^*P* < 0.01; ^∗∗∗^*P* < 0.001.

Wound-healing assays and transwell assays were performed to analyze the effect of ADRB2 on GIST cell migration and invasion. The wound healing assay results demonstrated that overexpression of ADRB2 enhanced the migratory ability of GIST cells, whereas silencing of ADRB2 significantly reduced the migratory ability (Figure 3(a)). Transwell assays also indicated that overexpression of ADRB2 increases the number of migrating and invading GIST cells, whereas knockdown of ADRB2 had the opposite effect on GIST cells (Figure 3(b)). Overall, these data suggested that ADRB2 promotes cell migration and invasion.

### 3.4. ADRB2 Reduces the Process of Apoptosis

Tumor cells become resistant to apoptosis, allowing them to survive longer. Flow cytometry analysis showed that silencing ADRB2 in GIST cells resulted in a significant increase in apoptotic cell death (Figure 3(c)) while the number of apoptotic GIST cells overexpressing ADRB2 was reduced compared to vector-transfected cells.

### 3.5. ADRB2 Promotes Tumor Proliferation and Metastasis *In Vivo*

**Figure 4:**
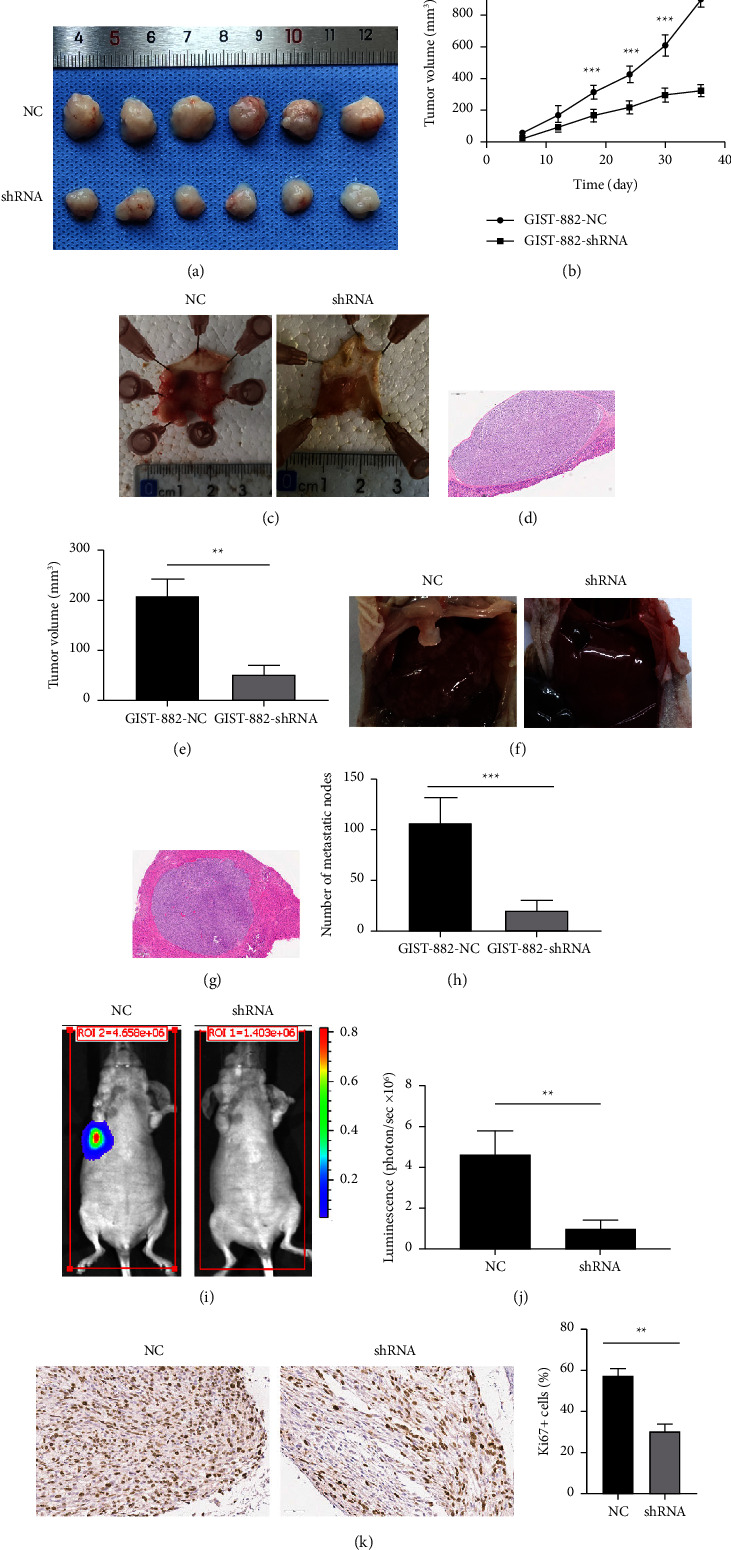
ADRB2 promotes the proliferation and metastasis of gastrointestinal stromal tumors in animal experiments. (a) Images of excised tumors from nude mice. (b) Tumor growth curves were used to evaluate the growth of xenograft tumors. (c) Representative images of gastric orthotopic tumors. (d) H&E staining of gastric orthotopic tumors. (e) Tumor volume of gastric orthotopic tumors. (f) Representative images of live metastasis form gastric orthotopic tumors. (g) H&E staining of liver metastasis. (h) Number of metastatic nodes. (i, j) In vivo image system was used to show the metastasis in intravenous injection model. (k) Ki67 expression was detected by IHC. Data are presented as the mean ± SD. ^∗∗^*P* < 0.01; ^∗∗∗^*P* < 0.001.

We performed a subcutaneous xenograft tumor model, an orthotopic gastric tumor model, and an intravenous injection model to evaluate the role of ADRB2 in vivo. In the subcutaneous xenograft tumor model, ADRB2-silenced cells had significantly reduced ability to form tumors in nude mice compared with the NC group (Figures 4(a) and 4(b)). Similar results were observed in the orthotopic gastric tumor model (Figures 4(c)–4(e)). In addition, we found that the orthotopic tumors developed fewer liver metastatic nodes in the ADRB2-knockdown group (Figures 4(f)–4(h)). Furthermore, ADRB2-knockdown also decreased lung metastatic burden in the intravenous injection model (Figures 4(i) and 4(j)). To confirm the ability of cell proliferation, we detect Ki67 in GIST tumors with IHC. As shown in Figure 4(k), knock down of ADRB2 significantly reduced ability of tumor cell proliferation compared with the NC group.

### 3.6. ADRB2 Enhances the ETV1-c-KIT Signaling

**Figure 5:**
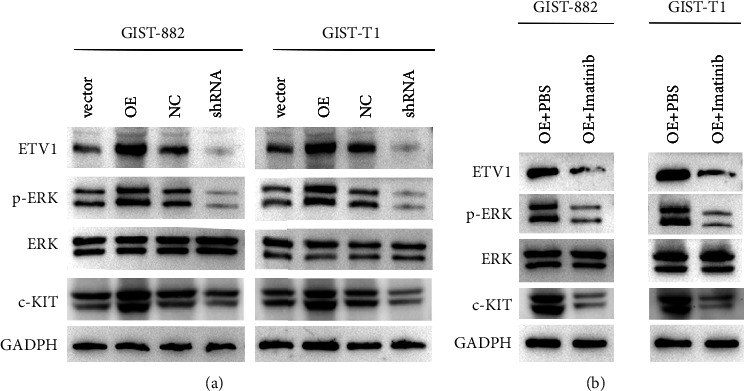
ADRB2 enhances the ETV1-c-KIT signaling. (a) Western blotting was used to detect the expression levels of ETV1, c-KIT, and p-ERK (Y204). (b) GIST cells were treated with or without imatinib mesylate, and ETV1, c-KIT, and p-ERK (Y204) expression levels were detected by western blotting.

**Figure 6:**
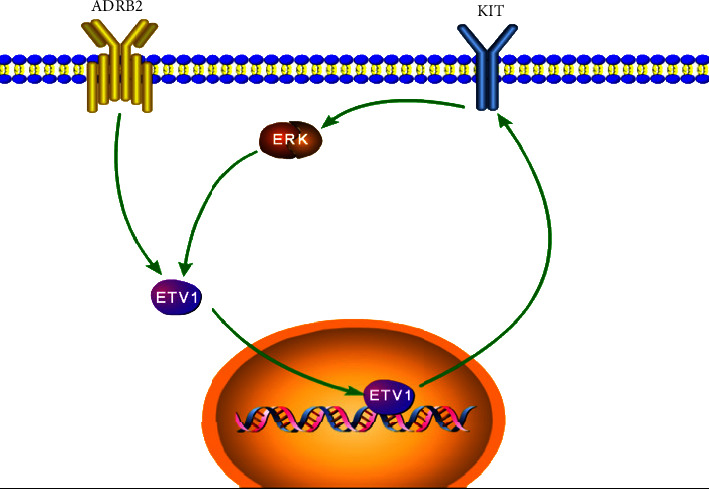
Schematic representation shows ADRB2 enhancing ETV1-c-KIT signaling by inducing ETV1.

We further explored the molecular mechanism of ADRB2 signaling-mediated effects. ETV1-c-KIT signaling is a key positive feedback to promote GIST progression [19]. In ADRB2-overexpressing GIST cells, ETV1, c-KIT, and p-ERK(Y204) expression were increased (Figure 5(a)). Silencing ADRB2 showed the opposite trend. When ADRB2-overexpressing GIST cells were inhibited by a c-KIT inhibitor (imatinib mesylate), it abolished the effect of ADRB2 on promoting ETV1, c-KIT, and p-ERK (Figure 5(b)). These results demonstrate that ADRB2 enhances the ETV1-c-KIT signaling by inducing ETV1 (Figure 6).

## 4. Discussion

In this study, we found that ADRB2 was highly expressed in 78.7% of GIST tissues and co-expressed with c-KIT by TMA and immunohistochemical staining. The expression levels of ADRB2 are significantly correlated with the clinical characteristics and prognosis of GIST patients. In vivo and in vitro experiments indicated that overexpression of ADRB2 promoted GIST proliferation and metastasis, while silencing ADRB2 expression showed the opposite trend. Taken together, ADRB2 is a potential target for the treatment of GIST.

In recent years, the influence of the nervous system on tumors has gradually attracted attention [20–22]. Several studies have found that neuron-like structures exist in tumor tissue [13, 23], and the density of nerve fibers is related to the degree of tumor differentiation [24]. Sympathetic nerves release neurotransmitters such as norepinephrine, which activates receptors and then causes downstream signal transduction by binding to specific receptors on the cell surface [25]. Previous studies have found that ADRB2 is the primary receptor mediating sympathetic signaling, and ADRB2 can activate multiple downstream signaling pathways in tumors [26–28]. The crosstalk of these different signaling pathways makes the single-targeted therapies currently used in the clinic more prone to failure. Therefore, it is essential to explore new therapeutic targets for the treatment of GIST.

The ETV1-c-KIT signaling is the key positive feedback to promote GIST progression [19]. Our study reveals that ADRB2 is essential for ETV1-c-KIT feedback in GISTs. Recently, ETV1 transcription factor was found to be a major regulator of GIST-specific transcription networks, and it is highly expressed in GISTs but not in other sarcomas. More importantly, ETV1 is upregulated by MAPK-ERK signaling, which is activated by c-KIT [29, 30]. In the present study, we found that the ERK pathway is activated by ADRB2, and ETV1 expression is increased in ADRB2-overexpression cells. The effects of ADRB2 on promoting ETV1-c-KIT signaling can be abolished by the c-KIT inhibitor imatinib. These data suggest that ADRB2 plays a vital role in GIST-specific transcription networks.

The expression levels of ADRB2 vary in different cancers. Here, we show that most GIST tissues have a high expression level of ADRB2. Besides, high levels of ADRB2 are correlated with risk level, tumor size, nuclear mitotic count, and liver metastasis. Kaplan–Meier survival analysis also shows that patients with high ADRB2 expression have a worse prognosis. These data indicate that ADRB2 might be a new target for GIST treatment. The sympathetic signaling can be abnormally activated by a variety of stimuli such as chronic stress. Activation of adrenergic signaling by chronic stress leads to sympathetic and adrenal medulla secretion of catecholamines [31, 32]. ADRB2 mediates most of the effects of catecholamines in tumors. Long-term elevated catecholamine levels can also have an impact on cancer progression [33]. Therefore, the combination of ADRB2 antagonists with existing GIST treatments may have clinical implications.

In conclusion, our study confirms that ADRB2 signaling promotes GIST proliferation and metastasis in vitro and in vivo. ETV1-c-KIT feedback is regulated by ADRB2-ERK signaling. ADRB2 is a potential prognostic marker and thus may serve as a new therapeutic target for GISTs.

## Figures and Tables

**Table 1:** Relations between ADRB2 expression and clinicopathologic characteristics of GIST patients who underwent radical surgery.

Clinicopathologic characteristics	*n*	ADRB2 expression		*χ* ^2^	*P*
		High	Low		
Ages (years)					
<60	71	59	12	1.970	0.161
≥60	51	37	14		

Gender					
Female	67	56	11	2.122	0.145
Male	55	40	15		

Tumor location					
Stomach	74	60	14	0.642	0.432
Other locations	48	36	12		

Risk level					
Very low/low	39	24	15	10.054	0.002
High/intermediate	83	72	11		

Tumor size					
<5 cm	50	33	17	8.134	0.004
≥5 cm	72	63	9		

Mitotic count (50 HPF)					
<5	71	49	20	5.578	0.018
≥5	51	47	6		

**Table 2:** Correlation between ADRB2 expression and liver metastasis.

Clinicopathologic characteristics	n	ADRB2 expression		*χ* ^2^	*P*
		High	Low		
Metastasis					
No	122	93	29	4.177	0.041
Yes	22	21	1		

**Table 3:** Correlation of ADRB2 expression with c-KIT level in GISTs.

ADRB2	c-KIT	No. of cases	*P*	*R* ^2^
−/+	−/+	15	<0.001	0.573
−/+	++	9		
−/+	+++	2		
++	−/+	7		
++	++	23		
++	+++	12		
+++	−/+	3		
+++	++	13		
+++	+++	38		

## Data Availability

The data used to support the findings of this study are included within the article.
